# Construction of a Full-Length cDNA Over-Expressing Library to Identify Valuable Genes from *Populus tomentosa*

**DOI:** 10.3390/ijms22073448

**Published:** 2021-03-26

**Authors:** Lingfei Kong, Zeyu Li, Qin Song, Xiaohong Li, Keming Luo

**Affiliations:** Chongqing Key Laboratory of Plant Resource Conservation and Germplasm Innovation, School of Life Sciences, Southwest University, Chongqing 400715, China; klf306037@126.com (L.K.); LLzeyuyeah@126.com (Z.L.); songqin518@126.com (Q.S.); l19961221@email.swu.edu.cn (X.L.)

**Keywords:** FOX hunting system, trichome development, secondary cell wall, *PtoCPCa*, *PtoWRKY13*

## Abstract

Poplar wood is the main source of renewable biomass energy worldwide, and is also considered to be a model system for studying woody plants. The Full-length cDNA Over-eXpressing (FOX) gene hunting system is an effective method for generating gain-of-function mutants. Large numbers of novel genes have successfully been identified from many herbaceous plants according to the phenotype of gain-of-function mutants under normal or abiotic stress conditions using this system. However, the system has not been used for functional gene identification with high-throughput mutant screening in woody plants. In this study, we constructed a FOX library from the Chinese white poplar, *Populus tomentosa*. The poplar cDNA library was constructed into the plant expression vector pEarleyGate101 and further transformed into *Arabidopsis thaliana* (thale cress). We collected 1749 T1 transgenic plants identified by PCR. Of these, 593 single PCR bands from different transgenic lines were randomly selected for sequencing, and 402 diverse sequences of poplar genes were isolated. Most of these genes were involved in photosynthesis, environmental adaptation, and ribosome biogenesis based on Kyoto Encyclopedia of Genes and Genomes (KEGG) pathway annotation. We characterized in detail two mutant lines carrying *PtoCPCa* or *PtoWRKY13* cDNA insertions. Phenotypic characterization showed that overexpression of these genes in *A. thaliana* affected trichome development or secondary cell wall (SCW) deposition, respectively. Together, the *Populus*-FOX-*Arabidopsis* library generated in our experiments will be helpful for efficient discovery of novel genes in poplar.

## 1. Introduction

Higher plants must resist the vagaries of different environments and their associated abiotic stresses, such as salinity, drought, and heavy metal toxicity, due to their sessile lifestyle and perennial growth habit [1,2]. Poplar spp. are considered to be excellent model plants for studying the mechanisms of secondary cell wall (SCW) deposition and wood development [3]. In addition to conventional breeding methods, large-scale screening of key functional genes in growth development and stress tolerance is also important for studying woody plants [4]. Unfortunately, there is a lack of genomic tools for identifying useful genes in different types of poplar.

In recent decades, loss-of-function mutant libraries constructed using T-DNA or transposon insertional mutagenesis approaches have been widely used in many plant species [5,6,7,8,9]. Numerous novel genes have been successfully cloned and characterized. However, due to the redundancy of genes, there may be only slight phenotypic differences between the wild-type (WT) and loss-of-function mutant plants [10,11,12]. Moreover, these methods usually generate recessive mutations, thus demanding a homozygote to display their phenotypes. Obviously, these methods cannot be applied to triploid tree species with long vegetative periods, such as hybrid *Populus tomentosa* (Chinese white poplar).

Gain-of-function mutation based on gene overexpression is an alternative and effective approach to evaluating gene function because it is unaffected by gene redundancy [13]. The gain-of-function mutant library has been developed using the activation tagging technique, which involves inserting tandem transcriptional enhancers into the plant genomes of many plant species, including *Populus* spp. [14,15,16,17]. By far the greatest number of valuable genes have been identified in poplar using this method [18,19,20,21]. The disadvantage of the activation tagging technique is that one enhancer can influence the transcription of genes on both sides of the insertion sites [22] and simultaneously increase the transcription of multiple genes, resulting in complex phenotypes. The Full-length cDNA Over-eXpressing (FOX) gene hunting system is one gain-of-function mutation approach, which can be used to avoid this problem [23]. Similar to the activation-tagging method, the FOX hunting system generates dominant mutations by overexpressing target genes [24]. Individual or limited numbers of normalized full-length cDNA segments from tissues or whole plants of interest under the control of the constitutive *CaMV 35S* promoter are directionally transformed into donor model plants, such as *Arabidopsis* spp. [24,25]. The ectopic expression of the full-length cDNAs may result in the expression of various phenotypes, which help to characterize the function of these candidate genes in plants [23]. To date, the FOX hunting system has been widely implemented for exploring gene function in *Arabidopsis* [23,26], rice [27,28], cotton [29], tomato [30], rape [31], and *Lotus* spp. [32], resulting in a high mutation rate, ranging from 10% to 20% [23,33].

To date, a large-scale FOX hunting system has not been generated in woody plants. However, recently, a xylem-specific mutant library of *Populus* was established based on this system [34]. According to the study, a total of only 113 transgenic lines were generated, whereas about 80% of these mutant lines were significantly affected in cellulose, lignin, and/or hemicellulose. Therefore, it is largely restricted to studying the function of most genes in poplars using this xylem-specific mutant library. In order to identify the helpful genes in all kinds of development processes and construct a genome-wide mutant library of *Populus,* in this study, we used the Gateway technology to generate a normalized genome-wide full-length cDNA library of *P. tomentosa* [35], and then the *Populus* FOX hunting library was transformed into *Arabidopsis thaliana* via the floral-dip method [36]. All of the transgenic plants were screened by phenotypic investigation. As a result, we collected 1749 positive T1 transgenic plants. Two mutant lines containing different poplar genes were selected for further functional exploration. Taken together, our results provide a rapid method for gene screening, which will provide insights into gene function in the complete genome of *P. tomentosa*.

## 2. Results

### 2.1. Construction of Populus-FOX-Arabidopsis Library

**Figure 1:**
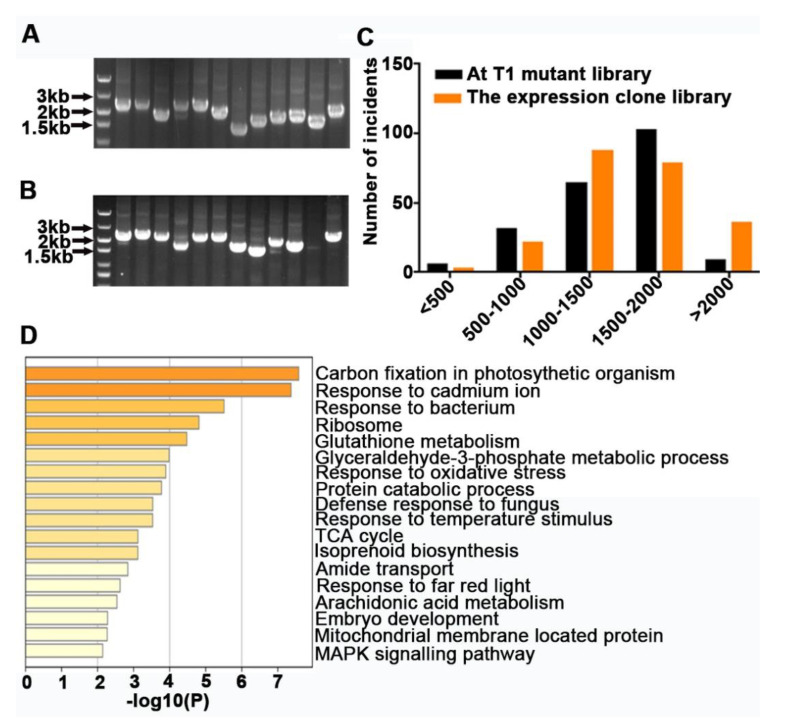
Construction of a genome-wide *Populus*-Full-length cDNA Over-eXpressing (FOX)-*Arabidopsis* library. (**A**) Size distribution of full-length cDNA transgenes in entry vector. (**B**) Size distribution of full-length cDNA transgenes in expression vector. (**C**) Sequence lengths of 200 randomly selected clones from the expression clone library and 200 cDNAs from T1 transgenic plants. (**D**) Functional annotation and classification according to Kyoto Encyclopedia of Genes and Genomes (KEGG) pathway assignments of full-length cDNA transgenes in *Arabidopsis thaliana*.

In order to eliminate the variance among individuals, we selected three independent WT plants of *P. tomentosa* to extract total RNA. After constructing the FOX library, 12 monoclonal colonies were randomly selected from the entry clone library and expression clone library and subjected to PCR determination for quality testing. As a result, the inserted fragment lengths ranged from 1.0 to 3.0 kilobases (kb) in both the entry and expression libraries (Figure 1A,B). Moreover, 11 colonies were positive for the insert, which is equivalent to a positive clone rate for the expression library of approximately 91.67% (Figure 1B). From the data, we can conclude that the expression library was effective.

To identify the transgenic plants, all T1 seedlings were sprayed with the herbicide Basta (phosphinothricin) (35% *v/v*) (Figure S1A,B). In total, we identified 2098 Basta-resistant plants, and 1749 of them were positive transgenic plants, which was confirmed by PCR determination (Figure S1C). Statistical analysis showed that the positive rate was up to 83.36%. We also compared the inserted fragment lengths randomly selected from transgenic plants with those from the expression clone library. The results showed that, in addition to the reduction in the enrichment of fragments longer than 2 kb in transgenic plants, the lengths of other fragments were proportional to those of the expression clone library (Figure 1C). To reveal the genes inserted into transgenic plants, a single PCR band was randomly selected for sequencing and isolated using a Basis Local Alignment Sequencing Tool (BLAST) search. The Kyoto Encyclopedia of Genes and Genomes (KEGG) Orthology database was used to summarize the function of these genes (Figure 1D). A total of 312 gene sequences were mapped to 47 pathways, related mainly to photosynthesis, environmental adaptation, ribosome biogenesis, glutathione metabolism, protein catabolic, embryo development, and mitogen-activated protein kinase (MAPK) signaling. In general, the *Populus*-FOX-*Arabidopsis* library has been successfully established.

### 2.2. Screening and Identification of Functional Genes in the FOX Library

**Figure 2:**
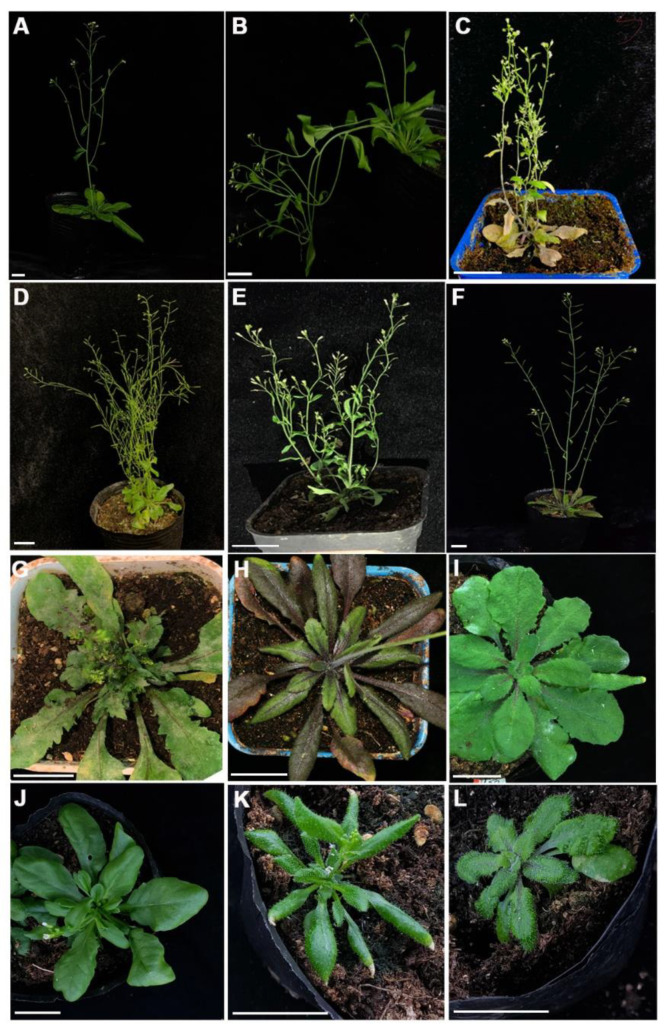
Morphological phenotypes of transgenic plants from the FOX library under normal conditions. (**A**) Wild-type (WT). (**B**) Lodging. (**C**) Early senescence and shorter siliques. (**D**) Smaller florescence leaves and more branches. (**E**) Poor growth and smaller siliques. (**F**) Stiffer stems. (**G**) Bigger leaves and later bolting. (**H**) Fewer branches and enhanced anthocyanin accumulation. (**I**) Thick leaves and later bolting. (**J**) Glabrous leaves. (**K**) Curled leaves. (**L**) Wrinkled leaves. Scale bars: (A–L) 2 cm.

In order to explore the genes affecting plant development via the FOX library, we observed and identified the phenotypes of the T2 generation from sequenced positive seedlings. The great majority of mutant plants showed distinct phenotypes, including lodging, dwarfing, increased branching, glabrous leaves, and so on (Figure 2A–L). Of the 402 FOX lines, 262 individuals showed obvious phenotypes. Interestingly, we found that many mutants exhibited multiple phenotypes, such as dwarfing associated with poor growth and small leaves associated with increased branching (Table 1), suggesting that these genes play important roles in plant growth and development.

**Figure 3:**
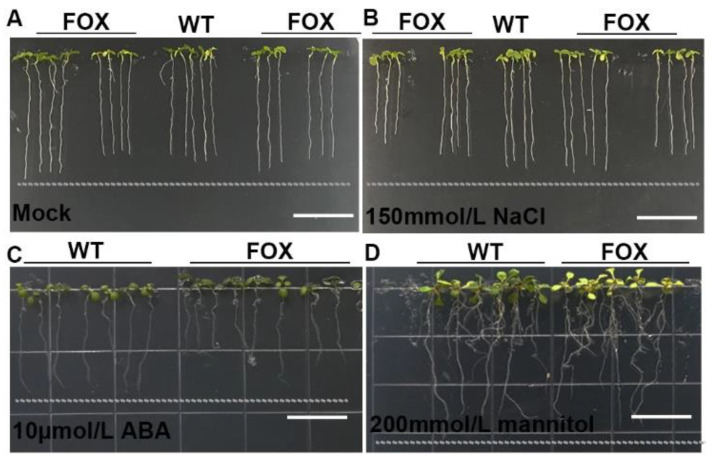
Screening of abiotic stress-resistant FOX mutants. (**A**) Mock. (**B**) 150 mmol/L NaCl treatment for 6 days. (**C**) 10 μmol/L ABA treatment for 6 days. (**D**) 200 mmol/L mannitol treatment for 10 days.

*P. tomentosa* is a perennial tree species with broad ecological adaptations. For the purpose of screening genes that are conducive to stress tolerance, we planted the seeds of T2 transgenic plants and inspected the phenotypic modulation in traits such as root length as well as seed germination under different chemical treatments (Figure S2). Under normal conditions, several FOX lines exhibited longer root lengths (Figure 3A). In 150 mM NaCl, compared with the WT, the root growth of some FOX plants was greatly affected, while the other FOX lines were still able to grow well (Figure 3B). In 10 μM abscisic acid (ABA), some FOX plants exhibited an ABA-hypersensitive phenotype (Figure 3C). In 350 mM mannitol, some FOX plants produced fewer lateral roots, and the root lengths were shorter, which was a typical drought-escape response (Figure 3D). As a result, we screened out 89 stress-responsive plants after processing.

### 2.3. Characterization of Two Mutants with Different Phenotypes to Evaluate the FOX Library

To verify that the phenotypic changes were the result of the ectopic expression of the corresponding inserted genes, we randomly selected two genes for further functional characterization after comparing different phenotypes and identifying homozygous genes.

**Figure 4:**
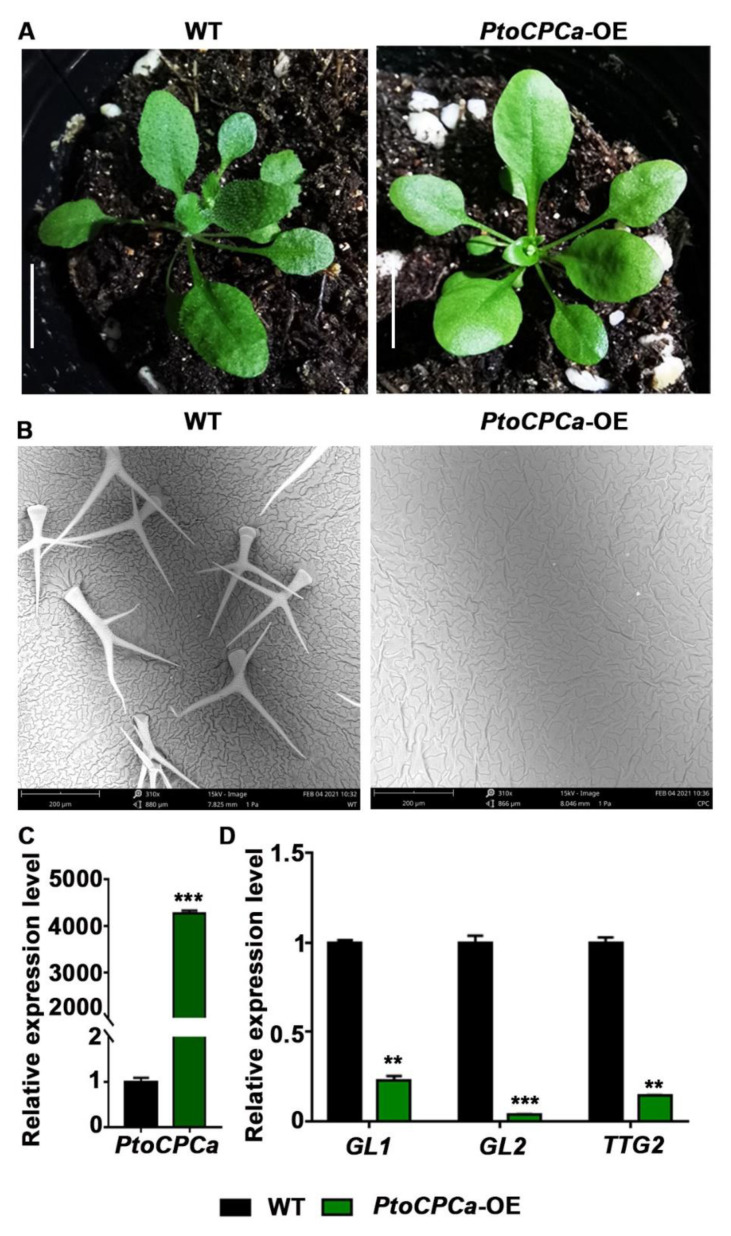
*PtoCPCa* overexpression inhibits trichome development in *A thaliana*. (**A**) Morphological phenotypes of 3-week-old WT and *PtoCPCa*-OE lines. (**B**) Scanning electron microscopy (SEM) of trichomes of 3-week-old WT and *PtoCPCa*-OE lines. (**C**) Expression levels of *PtoCPCa* in WT and *PtoCPCa*-OE lines. (**D**) Expression levels of trichome development marker genes in WT and *PtoCPCa*-OE lines, including *GL1* (AT3G27920), *GL2* (AT1G79840), and *TTG2* (AT2G37260). *Actin7* was used as an internal control. Error bars represent the standard error (S.E.) of three independent biological replicates. Asterisks indicate a significant difference with respect to WT plants using the Student’s *t*-test (**, *p* < 0.01; ***, *p* < 0.001). Scale bars: (**A**) 1 cm; (**B**) 200 μm.

In all of the transgenic plants with altered variable leaves, K103 showed unique glabrous leaves. BLAST searching suggested that the inserted gene was a homolog of Potri.002G168900. Amino acid sequence alignment and phylogenetic tree analysis showed that the gene shares a high degree of homology with *AtCPC* (Figure S3) in *A. thaliana*, so we named it *PtoCPCa*. Compared to the WT plants, seedlings that overexpressed *PtoCPCa* presented smooth glabrous leaves (Figure 4A). Quantitative measurement of leaf trichomes by scanning electron microscopy (SEM) revealed a significant reduction in trichome density. The trichome density of *PtoCPCa*-OE seedlings was nearly 0/mm^2^, while the density of WT seedlings was 10.33/mm^2^ (Figure 4B). Trichome initiation and development are modulated by a complicated network that includes hormone signaling (gibberellin, jasmonic acid) [37,38], a MYB-bHLH-WD40 complex hub comprising *GL1, TTG1, GL3*, and *EGL3* [39,40,41], as well as some key transcription factors, such as *TRY, CPC, GL2, TTG2,* etc. [42,43,44]. According to the qRT-PCR analysis we carried out, the relative expression level of the *PtoCPCa* gene was extremely high in the K103 lines (Figure 4C), while the levels of *GL1, GL2*, and *TTG2* were reduced in the *PtoCPCa*-OE lines compared to those of the WT plants (Figure 4D). Suppression of these key factors may have been the cause of aberrant trichome development. This is consistent with the results of a previous study of *Arabidopsis* [42]. In addition, *PtrTCL1* (homolog of *PtoCPCa*) ectopic expression in *Arabidopsis* also resulted in a similar phenotype [45]. This result suggested that our *Populus*-FOX-*Arabidopsis* library is an effective method for exploring functional genes.

**Figure 5:**
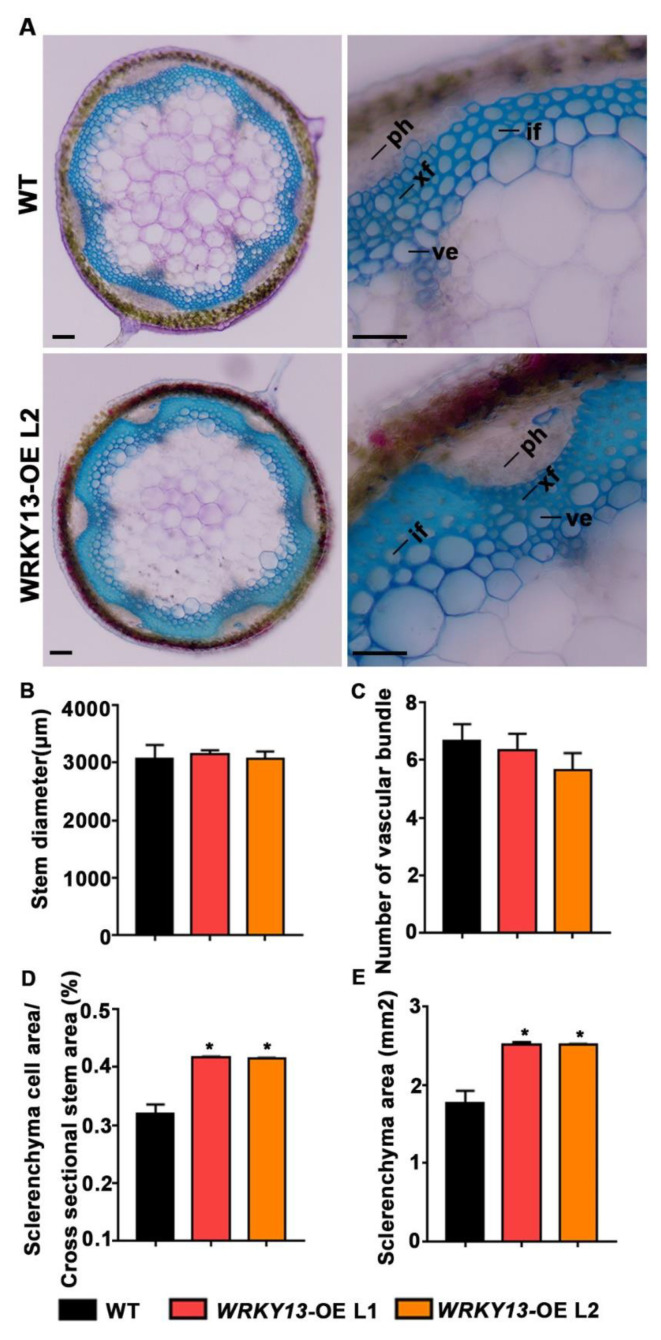
*PtoWRKY13* overexpression promotes secondary cell wall (SCW) deposition in transgenic *A. thaliana* inflorescence stems. (**A**) Cross-sectioning and staining with Toluidine Blue of basal stems of 6-week-old WT and *PtoWRKY13*-OE *A. thaliana*. (**B**) Stem diameter of WT and *PtoWRKY13*-OE lines. (**C**) Number of vessel bundles in WT and *PtoWRKY13*-OE lines. (**D**) Percentage of sclerenchyma cells in the stems of WT and *PtoWRKY13*-OE lines. (**E**) Sclerenchyma area in the stems of WT and *PtoWRKY13*-OE lines. (*, *p* < 0.05); ph, phloem; xf, xylem fiber; ve, vessel; if, interfascicular fiber. Scale bars: (**A**) 100 μm.

**Figure 6:**
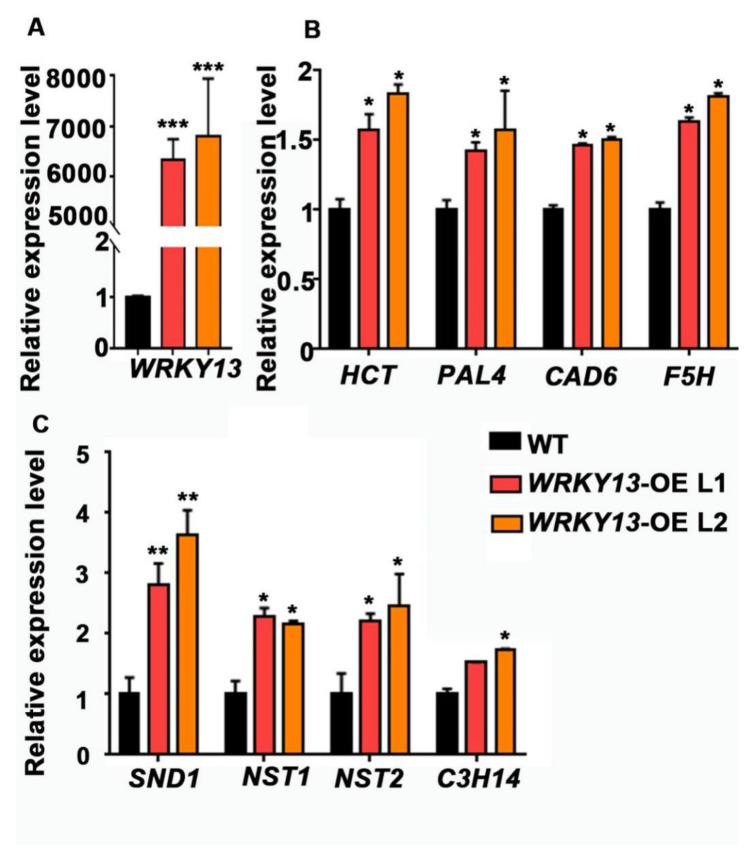
Lignin biosynthetic genes and secondary wall-related genes are altered in *PtoWRKY13*-OE lines. (**A**) Expression levels of *PtoWRKY13* in WT and *PtoWRKY13*-OE seedlings. (**B**) Expression levels of lignin pathway genes *HCT* (AT5G48930), *PAL4* (AT3G10340)*, CAD 6*(AT4G37070), and *F5H* (AT4G36220) in WT and transgenic *A. thaliana*. (**C**) Expression levels of secondary wall-related key transcription factors *SND1* (AT1G32770), *NST1* (AT2G46770), *NST2* (AT3G61910), and *C3H14* (AT1G66810) in WT and transgenic *A. thaliana*. *Actin7* was used as an internal reference gene. Error bars represent the standard error (S.E.) of three independent biological replicates. Asterisks indicate a significant difference with respect to WT plants using the Student’s *t*-test (*, *p* < 0.05; **, *p* < 0.01; ***, *p* < 0.001).

We also wondered whether our FOX library could hunt out some novel genes. Indeed, according to the annotations, there are still some genes with unknown functions (Table 1). We found two individual mutant lines (K23 and K78) displaying the parallel phenotype of stiffer inflorescence stems, and both of them had the same gene Potri.005G086400 inserted, which encodes a WRKY transcription factor that had not previously been reported in poplar. Phylogenetic analysis indicated that Potri.005G086400 is the homologous gene of *AtWRKY13* (Figure S4A), and we named it *PtoWRKY13*, based on a previous study [46]. Amino acid sequence alignment suggested that, except for a conserved WRKY domain, only a few amino acid residues showed no difference between *PtoWRKY13* and *AtWRKY13* (Figure S4B). It has been reported that *AtWRKY13* affects SCW deposition by directed modulation of *NST2* in *Arabidopsis* [47]. After staining with Toluidine Blue, observation of the basal cross-sections of the inflorescence stems from 6-week-old plants showed that, although *PtoWRKY13*-OE lines exhibit similar stem diameters and numbers of vascular bundles (Figure 5B,C), the SCWs were exaggeratedly thicker than those of WT plants (Figure 5A,D,E). We first detected the expression level of *PtoWRKY13* in the two mutants by qRT-PCR analysis, and the results showed that both FOX lines had a high expression level of *PtoWRKY13* (Figure 6A). In *A. thaliana*, the *wrky13* mutant showed the phenotype of lignin reduction. Hence, we examined the expression of SCW biosynthetic genes in the *PtoWRKY13-OE* lines. The expression levels of four lignin pathway genes (*HCT, PAL4, CAD6*, and *F5H*) and four transcription factors (*SND1, NST1, NST2*, and *C3H14*) were all up-regulated in the *PtoWRKY13*-OE lines (Figure 6B,C). The results were fascinating because of the great differences between the amino acid sequences of *PtoWRKY13* and *AtWRKY13*. It is interesting to further verify the phenotype and regulatory mechanism of *PtoWRKY13* in poplar, as it may prove to be an important factor in improving the properties of poplar wood.

## 3. Discussion

Gene function studies rely on the creation of mutant materials under existing experimental conditions. Up until now, the whole genome loss-of-function mutant libraries have successfully been developed in several plant species, including *A. thaliana* and rice, which greatly enable the discovery of functional genes [6,48]. With regard to polyploid plants, loss-of-function mutant generation via T-DNA, transposon insertion, and physical and chemical mutagenesis usually deprives the function of a limited number of genes in multigene families. The other members of the family may play similar roles in rescuing the function, so that the mutant appears to possess no observed phenotype. Furthermore, T-DNA insertion is a random event that occurs on the genome, which makes it difficult to screen out helpful mutants. Therefore, gain-of-function mutagenesis is a better choice for studying polyploid plants, of which the FOX hunting system is a representative technique. Compared with the loss-of-function method, it is convenient to discover the inserted genes from the FOX hunting mutant by PCR amplification and sequencing using universal primer pairs with no need for positional cloning or TAIL-PCR [23,27]. Moreover, the FOX system is a versatile tool for constructing a genome-wide library or specific development process-related library [26,34] due to the fact that the number of cDNAs in the library can be altered as needed. The screening of the FOX library can also be undertaken in conditions of interest, for example, high Zn concentrations [49], high salinity, or drought [29]. To date, the FOX hunting system has been widely used in polyploid plants such as cotton and rape [29,31]. These studies suggest that FOX is an effective method for exploring functional genes.

In this study, the genome-wide full-length cDNA of *P. tomentosa* was cloned to the pEarleyGate101 plant expression vector containing the Basta-resistant gene using the high-throughput Gateway technique. This FOX library was then transformed into *A. thaliana* to facilitate efficient screening of functional genes. As a result, we transformed more than 10,000 WT *A. thaliana* plants and obtained 2098 Basta-resistant plants, including 1749 positive transgenic plants by exploiting the Bar gene on the pEarleyGate100 binary expression vector as a selection marker and PCR identification, respectively (Supplementary Figure S1B,C). The positive rate was up to 83.36%, and the inserted fragment lengths ranged from 1to 3 kb. These results were consistent with those of previous studies using this technique. For example, the inserted fragments of a cotton-FOX-*Arabidopsis* overexpression library varied from 0.5 to 3.0 kb [29]; the average length of fragments from an *Oryza*-FOX-*Arabidopsis* plant was 1.5–2.0 kb [33]; in *A. thaliana*, the length ranged from 0.3 to 3.0 kb [23].

Rapidity and validity are two important metrics for a gene discovery technique. In our experiments, various phenotypes were observed, including lodging, wrinkled leaves, greater branching, and so on in the T1 generation (Figure 2A–L). Most of the phenotypes were inserted into a signal fragment, confirmed by PCR amplification. The results above indicated that we screened out a series of functional genes using the FOX system. Indeed, functional characterization of a randomly selected K103 line found that the phenotype was caused by overexpression of the corresponding inserted gene. K103 showed glabrous leaves, and blast searching and phylogenetic analysis demonstrated that *PtoCPCa* was inserted into K103. As shown in Figure 3D, the transcription levels of the trichome development key factors *GL1, GL2*, and *TTG2* were reduced. This result is consistent with those of previous studies of *A. thaliana* [42], and were verified by experiments on *P. trichocarpa* [45]. Trichome development was affected by overexpression of eight poplar R3-MYB genes (including *PtrCPCa*, which was named *PtrTCL1*). Interestingly, one of the trichome development key transcription factors, *AtGTL1*, was first identified by FOX library screening [50], indicating that the FOX hunting system might be the unique method to identify various key factors from the specific development regulation network. In conclusion, we successfully screened out numerous functional genes via our FOX library.

Except for genes that had already been reported, we also hunted for a large number of novel genes. *PtoWRKY13* can be considered a representative of genes with unknown functions. The formation of wood is an important and characteristic function of poplar, and the development of wood comes from the differentiation of the cambium cells into xylem cells, which then go on to cell expansion, SCW deposition, programmed cell death, and eventually wood formation [51]. The properties of wood are affected mainly by the deposition of SCWs. It is obvious that some genes of the NAC family play master switch roles in the regulation of SCW formation [52,53], while the existence of regulators that function upstream remains unknown. According to various studies, a few WRKY transcription factor family members play such a role. It has been reported that *AtWRKY12* initiates pith SCW formation by directly regulating *NST2* [54], and *AtWRKY13* functions as an activator to directly regulate *NST2* [47]. Additionally, *AtWRKY15* suppresses tracheary element differentiation by regulating the upstream element VND7, possibly via auxin signaling [55]. The regulation of auxin in SCW formation has been widely reported [56,57] and might operate mainly through *ASYMMETRIC LEAVES2 (AS2)/LATERAL ORGAN BOUNDARIES DOMAIN (LBD)* family proteins. *LBD29*, which involves auxin signaling, represses the NAC master regulators and fiber wall biosynthesis [58], while *LBD30/18* genes act downstream of ARF7 and are involved in a positive feedback loop for VND7 expression [59]. *PtoWRKY13* overexpression lines K25/K78 showed stiffer stems and thickened SCWs (Figure 6A). Levels of lignin biosynthetic genes, including *HCT, PAL4, CAD6*, and *F5H*, were increased as well as those of SCW master switches *SND1, NST1*, and *NST2* (Figure 6B,C). However, it is still not clear whether *PtoWRKY13* directly regulates WNDs and the relationship with LBD proteins in *P. tomentosa*. In order to solve this problem, transgenic poplar will be generated in order to verify the phenotype and reveal the molecular mechanism in future studies.

## 4. Materials and Methods

### 4.1. Plant Materials and Growth Conditions

*Populus tomentosa* Carr. (clone 741) were grown in the greenhouse at 22 °C with a 16/8 h light/dark cycle with supplemental light (4000 lux), and the relative humidity should be around 60% for the optimum growth condition. *Arabidopsis thaliana* (Col-0) seedlings were grown in an incubator at 22 °C with 16 h 10,000 lux light and 8 h dark. The relative humidity was kept at 60% for *P. tomentosa* and 80% for *Arabidopsis thaliana*.

In order to observe the difference in sensitivity to salt stress during seed germination, seeds were evenly placed on MS medium supplemented with 150 mM NaCl. In order to observe the difference in sensitivity to stress during seedlings growth, seeds were evenly placed on MS medium supplemented with 3% (*w*/*v*) sucrose and 10 μM ABA, 150 mM NaCl, or 200 mM mannitol. Statistical germination rates were calculated from 1 to 9 days after the earliest germination. Root lengths were measured after 10 days of vertical culture.

The seeds of WT Col-0, homozygous *PtoCPCa*, and *PtoWRKY13* overexpressing lines were kept at 4 °C for 3 days before being placed on MS medium supplemented with 3% (*w*/*v*) sucrose. After 10 days of germination on MS plates, the seedlings were transferred into soil and developed in a growth incubator at 22 °C under long-day conditions (16 h light/8 h dark).

### 4.2. RNA Extraction

Total RNA of poplar was extracted and purified using RNA RNeasy Plant Mini Kit (Qiagen, Hilden, Germany). The OD260/OD280 ratio measured via a NanoDrop 2000 Spectrophotometer (Thermo, West Palm Beach, FL, USA) and electrophoresis were used to judge the RNA quality. Total RNA (2 µg) treated with RNase-free DNase (Takara Dalian, China) was subjected to first-strand cDNA synthesis in a total volume of 20 µl using RT-AMV transcriptase (Takara, Dalian, China).

### 4.3. Construction of P. tomentosa Fl-cDNA Overexpression Library, Plant Transformation and Selection

*Populus* full-length cDNA was synthesized by reverse transcription with the isolated mRNA. DSN (Duplex-Specific Nuclease) enzyme was used to normalize the concentration of cDNA to generate a normalized FLcDNA library. The cDNA library was firstly cloned into an entry vector pDONR222, and then into an pEarleyGate101 plant expression vector using high-throughput Gateway technology

*Populus*-FOX-*Arabidopsis* library was produced by using the Gateway cloning technology [35] (Invitrogen, Carlsbad, CA, USA). Gateway technology contains two steps: BP recombination reaction (PCR fragment+ entry vector) and LR recombination reaction (entry vector after BP recombination reaction+ destination vector). After LR recombination reaction, the cDNAs were recombined into overexpression vector pEarleyGate101 (Invitrogen, Carlsbad, CA, USA), and the resulting construct resistance to glufosinate was introduced into *Agrobacterium tumefaciens* strain GV3101 by the freeze–thaw method.

The expression vector library was then transformed into *Agrobacterium tumefaciens* strain GV3101 and introduced into WT *Arabidopsis* seedlings using floral-dip method [36]. Subsequently, leaves selected from Basta-resistant T1 plants were used for DNA extraction and PCR analysis to confirm cDNA diversity. Transformed T1 seeds were selected with 350 mg/L of Basta solution. Phenotypes were scored based on morphological changes such as germination, leave size, shape, and color. All plants showing visible phenotypes were transferred to a new growing tray.

### 4.4. Genomic DNA Isolation, PCR and Sequencing

To identify integrated cDNAs, genomic DNA prepared from leaves of randomly selected 2100 T1 transgenic plants was the template for PCR amplification with primers complementary to vector sequences flanking the attB1 and attB2 sites. The PCR condition was 94 °C for 30 s for denaturation, 57 °C for 30 s for annealing and 72 °C for 120 s for elongation. The PCR products were gel purified and sequenced with the same primers. The identity of the transcript was revealed by sequence homology search using the Phytozome BLAST tool. To validate the phenotype conferred by the inducible expression of fl-cDNA, the cDNA was isolated and inserted into pEarleyGate101 expression vector driven by 35S promoter for Agrobacterium-mediated transformation of Arabidopsis wild-type (Col-0).

### 4.5. Phylogenetic Analysis and Sequence Alignment

A phylogenetic tree was developed with MEGA6 (Lynnon Biosoft, Quebec, QC, Canada) using the neighbor-joining method (Tamura et al. 2013). Multiple alignments of protein sequences were conducted with the software DNAMAN 7 (Lynnon Biosoft, San Ramon, CA, USA).

### 4.6. Gene Expression Analysis

For quantitative real time PCR (qRT-PCR) analysis, total RNA from leaf tissues or stem tissues was extracted using RNA RNeasy Plant Mini Kit (Qiagen, Hilden, Germany) and treated with RNase-free DNase (TaKaRA, Dalian, China). Samples from at least three plants were pooled for analysis. The quality or integrity of RNA was checked by agarose gel electrophoresis and P100 spectrophotometer (Pultton, Ann Arbor, MI, USA). The criteria of high-quality total RNA include the following: (1) sharp distinct 28S and 18S rRNA bands, with the 28S band approximately twice as intense as the 18S band; (2) the value of D260/OD280 between 1.9 and 2.0; and (3) no detected genomic DNA band. The qualified RNA samples were reversely transcribed using RT-AMV (Avian Myeloblastosis Virus) transcriptase (TaKaRa, Dalian, China). Subsequently, qRT-PCR was performed in a volume of 20 μL containing 10 μL of SYBR Premix ExTaq TM (TaKaRa, Dalian, China). *ACTIN7* and *UBQ* were used as an internal control in *Arabidopsis* and *Populus*, respectively. Three biological replicates of each sample and three technical replicates of each biological experiment were conducted. Primers used for qRT-PCR were listed in Table S1.

### 4.7. Microscopy and Histochemistry

The basal inflorescence stems of 6-week-old WT and transgenic *Arabidopsis* seedlings were cross-sectioned by using a Vibrating blade microtome (VT1000S, Leica, Weitzlar, Germany). To detect lignified cell walls, stem cross-sections (100 μm thickness) were stained with Toluidine Blue-O (TBO, 0.5% *w/v*). Observations were performed using an Olympus BX53 microscope.

### 4.8. Scanning Electron Microscopy (SEM) Observation

The abaxial epidermis of 3-week-old WT and *PtoCPCa*-OE transgenic Arabidopsis leaves were attached to sample stage using double-sided sticky tape and then directly viewed under a scanning electron microscope (Phenom™ Pure FEI, Eindhoven, The Netherland). The images were captured digitally. At least three biological replicates were measured for each test.

## 5. Conclusions

In this study, a genome-wide full-length *Populus*-FOX-*Arabidopsis* overexpression library was constructed using Gateway technology. A total of 1749 positive transgenic plants were obtained and various phenotypes were observed, such as lodging, poor growth, dwarfism, and so on in the T1 generation. Genes screened out from the library were related mainly to photosynthesis, environmental adaptation, and ribosome biogenesis based on KEGG pathway annotation. A total of 89 stress-responsive plants were screened out under NaCl, mannitol, or ABA conditions. This library provides a novel resource for exploring genes related to perennial growth and abiotic stress tolerance. To evaluate the FOX library, two mutant lines carrying *PtoCPCa* or *PtoWRKY13* cDNA insertions were selected for further functional exploration in trichome development and secondary wall deposition respectively. The results demonstrated that our FOX library could be used to identify valuable genes from various development processes. In brief, our study showed a rapid gene discovery method that will provide important insights into the gene function of poplar in the whole genome. Future work will be focused mainly on novel gene characterization and molecular mechanism analysis.

## Figures and Tables

**Table 1:** Phenotypes and functional annotation of mutant FOX cDNAs.

Line	*Pt* Gene ID	*At* Gene ID	Query Cover	Phenotype	Annotation
N1	Potri.002G212700.1	AT1G65410.1	0.728	Poor growth, thin stem	NON-INTRINSIC ABC PROTEIN 11 (ATNAP11)
N3	Potri.005G175900.1	AT1G44835.2	0.715	Poor growth, thin stem	YbaK/aminoacyl-tRNA synthetase-associated domain-containing protein
N46	Potri.008G075200.1	AT3G10985.1	0.602	Poor growth, senilism	SENESCENCE ASSOCIATED GENE 20 (ATSAG20)
A33	Potri.006G224000.1	AT5G26180.1	0.7	Dwarf	TRNA METHYLTRANSFERASE 4H (ATTRM4H)
N66	Potri.006G130900.1	AT5G60390.3	0.953	Dwarf	GTP binding Elongation factor Tu family protein
N4	Potri.003G126100.1	AT4G11600.1	0.677	Dwarf, poor growth	GLUTATHIONE PEROXIDASE 6 (ATGPX6)
N9	Potri.010G013400.1	AT3G05540.1	0.792	Dwarf, poor growth	TRANSLATIONALLY CONTROLLED TUMOR PROTEIN 2 (ATTCTP2)
N15	Potri.012G114200.1	AT5G61670.2	0.772	Dwarf, Poor growth	Protein disulfide-isomerase/S-S rearrangase
N10	Potri.001G123200.1	AT4G16530.1	0.453	Dwarf, poor growth	DUF577
N19	Potri.018G119600.1	AT1G75400.1	0.354	Dwarf, delayed flowering	RING/U-box superfamily protein
N13	Potri.011G045100.1	AT2G20840.1	0.788	Dwarf, delayed flowering	SECRETORY CARRIER MEMBRANE PROTEIN 3 (ATSCAMP3)
N11	Potri.008G145600.1	AT1G17960.1	0.542	Dwarf, fewer branches	Threonyl-tRNA synthetase
N6	Potri.007G011200.1	AT4G37870.1	0.873	Dwarf, fewer branches	PHOSPHOENOLPYRUVATE CARBOXYKINASE 1 (ATPCK1)
N40	Potri.004G233800.1	AT5G61510.1	0.753	Fewer branches	GroES-like zinc-binding alcohol dehydrogenase family protein
N65	Potri.019G058000.1	AT3G56360.1	0.542	Fewer branches	Hypothetical protein
A5	Potri.008G178700.1	AT1G24360.1	0.693	Rapid growth	NAD(P)-binding Rossmann-fold superfamily protein
A11	Potri.016G091200.1	AT5G03290.1	0.89	Rapid growth, big leaves	ISOCITRATE DEHYDROGENASE V (ATIDHV)
A4	Potri.012G047200.1	AT1G73590.1	0.823	Slender petioles	PIN-FORMED 1 (ATPIN1)
A1	Potri.017G153500.1	AT1G17860.1	0.665	Slender petioles, dwarf	THALIANA KUNITZ TRYPSIN INHIBITOR 5 (ATKTI5)
A2	Potri.001G162800.1	AT1G17290.1	0.841	Slender petioles, small leaves	ALANINE AMINOTRANSFERASE (ATAlaAT1)
A12	Potri.019G054600.1	AT1G24706.2	0.742	Slender petioles	THO/TREX complex
A7	Potri.016G084800.1	AT3G09640.1	0.86	Small leaves, more branches	ASCORBATE PEROXIDASE 2 (ATAPX2)
N21	Potri.004G177500.1	AT4G38510.4	0.98	Small leaves, more branches	V-ATPASE B SUBUNIT 2 (ATVAB2)
A3	Potri.009G042400.1	AT3G54140.1	0.833	Small leaves, more branches	PEPTIDE TRANSPORTER 1 (ATPTR1)
A6	Potri.005G242500.1	AT5G42150.1	0.775	Small leaves	Glutathione S-transferase family protein
A10	Potri.009G133000.1	AT4G38630.1	0.617	Small leaves	MULTIUBIQUITIN-CHAIN-BINDING PROTEIN 1 (ATMCB1)
N23	Potri.008G056300.1	AT3G55440.1	0.744	Fewer cauline leaves	CYTOSOLIC TRIOSE PHOSPHATE ISOMERASE (ATCTIMC)
N18	Potri.015G029000.1	AT1G48410.1	0.915	Serrated leaves, delayed flowering, malformed siliques	ARGONAUTE 1 (ATAGO1)
N94	Potri.002G015100.1	AT3G03190.1	0.642	Small leaves, thin stem	GLUTATHIONE S-TRANSFERASE F11 (ATGSTF11)
N96	Potri.001G413500.1	AT2G31670.1	0.649	Thin stem	Stress responsive alpha-beta barrel domain protein.
N73	Potri.004G015300.1	AT4G21960.1	0.843	Stiffer stem	Peroxidase superfamily protein
A9	Potri.008G161200.1	AT4G14550.1	0.756	Thicker stem	INDOLE-3-ACETIC ACID INDUCIBLE 14 (ATIAA14)
N8	Potri.005G146800.1	AT5G67500.1	0.83	Lodging	VOLTAGE DEPENDENT ANION CHANNEL 2 (ATVDAC2)
A14	Potri.004G081700.1	AT1G27190.1	0.819	Seed abortion	BAK1-INTERACTING RECEPTOR-LIKE KINASE 3 (ATBIR3)

Query cover based on BLAST analysis against the *Populus trichocarpa* V3.0 database on phytozome. *At—Arabidopsis thaliana*, *Pt—P. trichocarpa.*

## Data Availability

Not applicable.

## Supplementary Materials

The following are available online at https://www.mdpi.com/article/10.3390/ijms22073448/s1. Figure S1: Screening and identification of positive FOX transgenic *Arabidopsis thaliana* seedlings. Figure S2: Screening of salt tolerance FOX mutants. Figure S3: Phylogenetic analysis and amino acid alignment of *PtoCPCa* and other R3 MYB transcription factors from different species. Figure S4: Phylogenetic analysis and amino acid alignment of *PtoWRKY13* and other WRKY transcription factors from different species. Supplementary Data Set 1: Primers used in this article.
