# Supplementary figures and images for: Construction of a Full-Length cDNA Over-Expressing Library to Identify Valuable Genes from Populus tomentosa
Source: Int J Mol Sci. 2021 Mar 26;22(7):3448. doi: 10.3390/ijms22073448 (PMC8036549; doi:10.3390/ijms22073448)

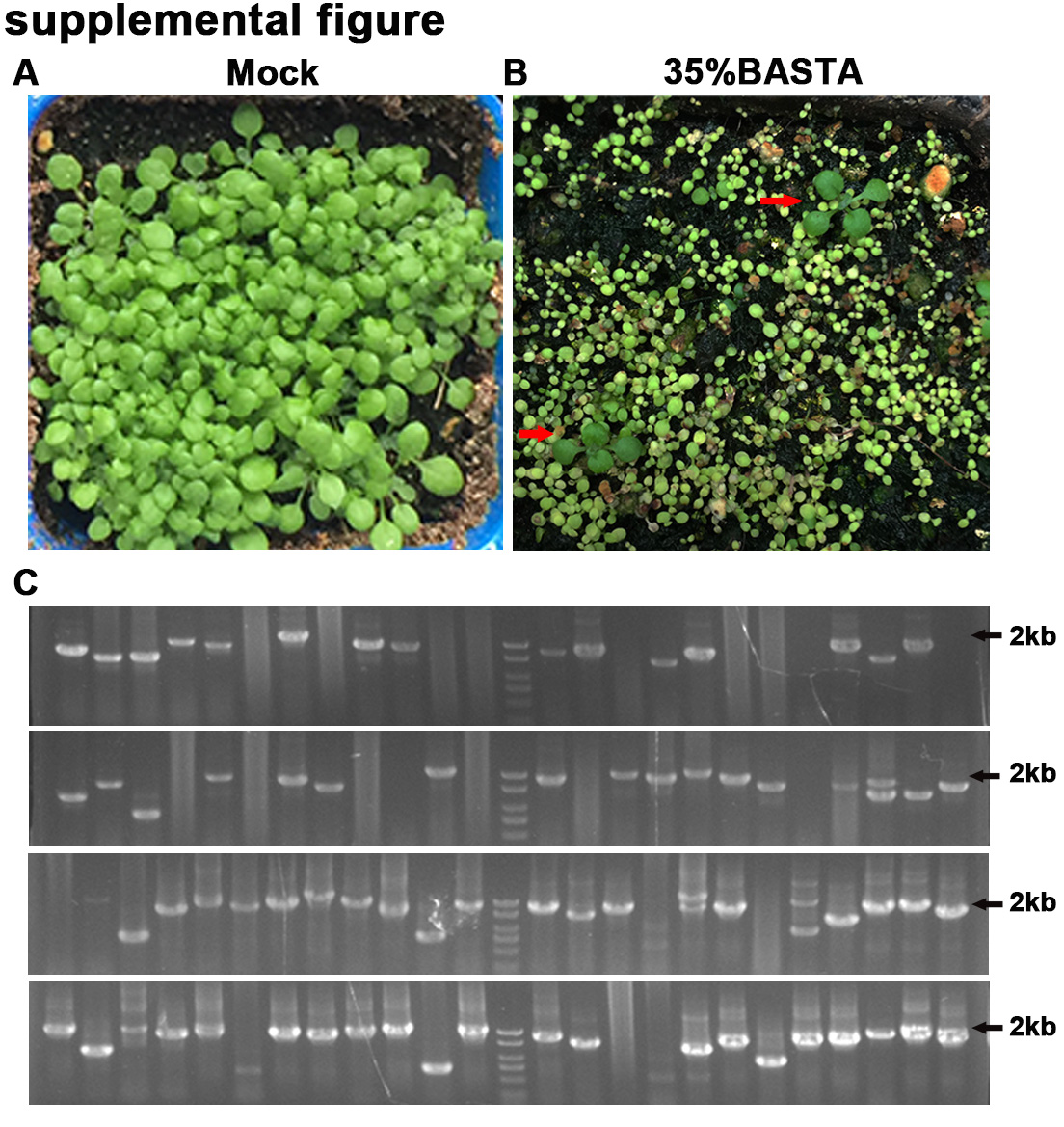

Supplement: Supplementary file 1 [file ijms-22-03448-s001.zip › Supplementary data/Figure S1.jpg]

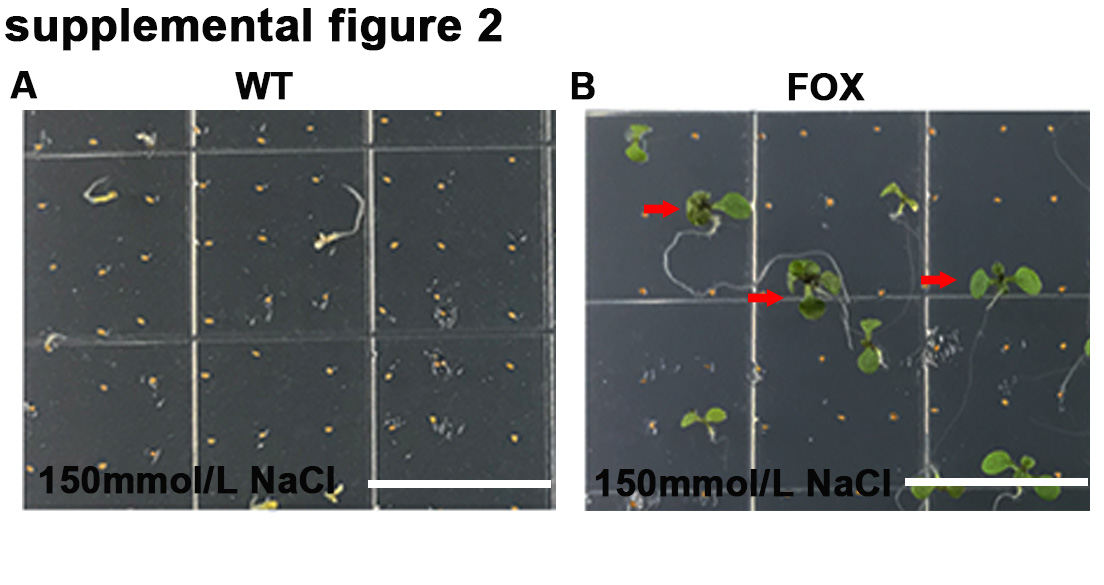

Supplement: Supplementary file 1 [file ijms-22-03448-s001.zip › Supplementary data/Figure S2.jpg]

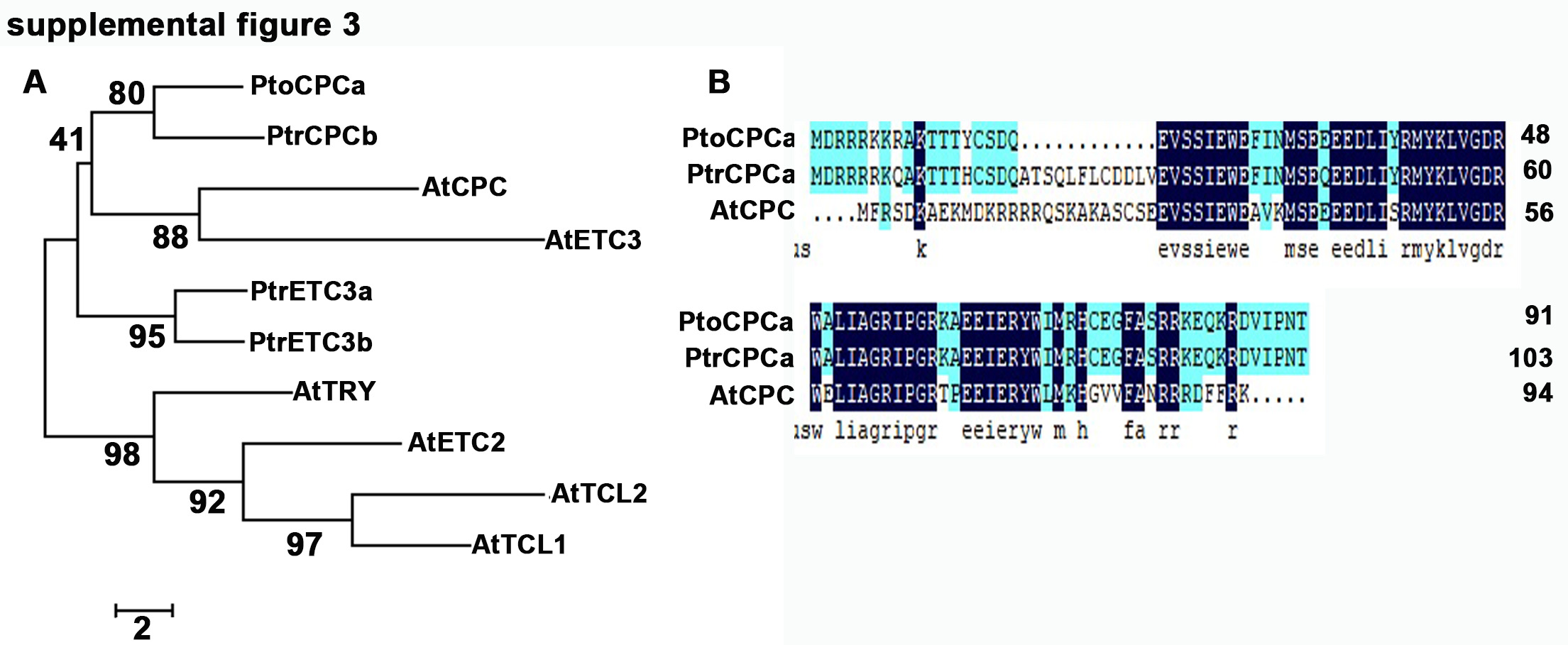

Supplement: Supplementary file 1 [file ijms-22-03448-s001.zip › Supplementary data/Figure S3.jpg]

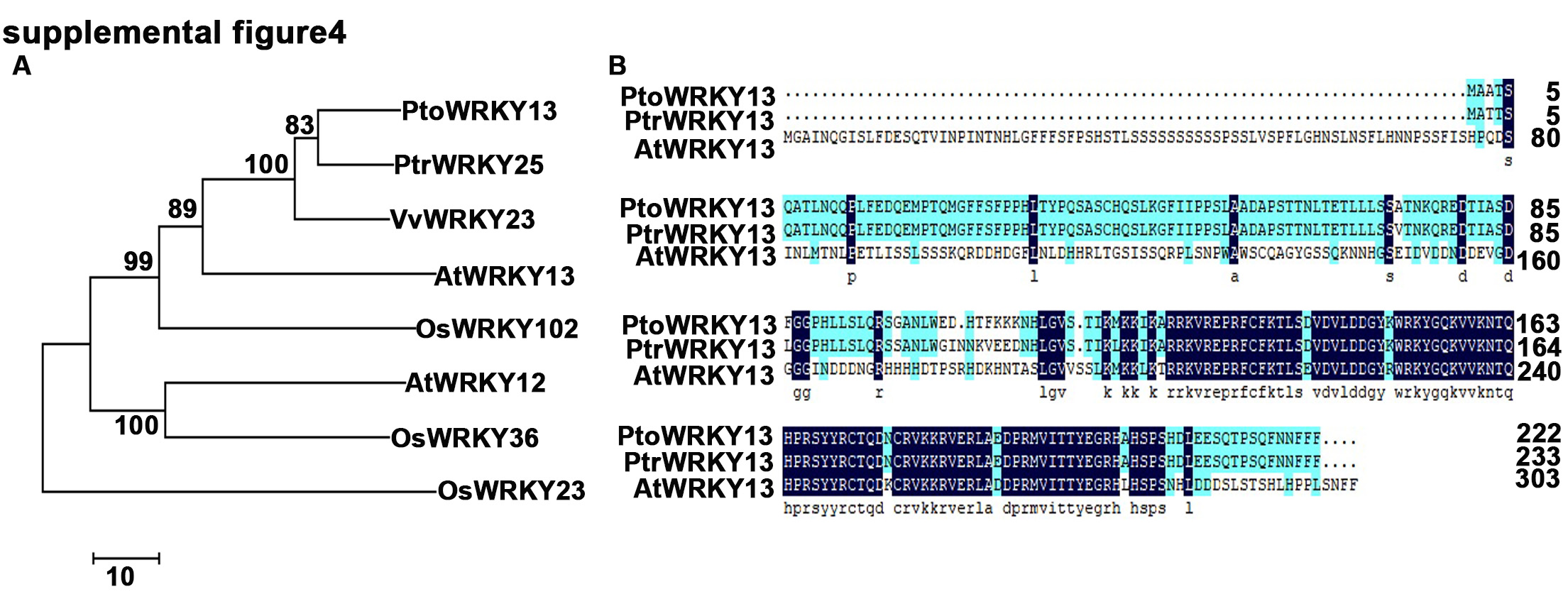

Supplement: Supplementary file 1 [file ijms-22-03448-s001.zip › Supplementary data/Figure S4.jpg]
